# Female-specific pharmacotherapy in ADHD: premenstrual adjustment of psychostimulant dosage

**DOI:** 10.3389/fpsyt.2023.1306194

**Published:** 2023-12-13

**Authors:** M. de Jong, D. S. M. R. Wynchank, E. van Andel, A. T. F. Beekman, J. J. S. Kooij

**Affiliations:** ^1^PsyQ, Expertise Centre Adult ADHD, The Hague, Netherlands; ^2^Department of Psychiatry, Amsterdam UMC/VUmc, Amsterdam, Netherlands; ^3^Amsterdam Public Health Research Institute, VU Medical Centre, Amsterdam, Netherlands; ^4^GGZ inGeest, Amsterdam, Netherlands

**Keywords:** attention deficit/hyperactivity disorder, female, women, pharmacotherapy, menstrual cycle, sex hormones, premenstrual, female specific therapy

## Abstract

**Objective:**

Attention-Deficit/Hyperactivity Disorder (ADHD) is a common neurodevelopmental condition which is underdiagnosed and undertreated in women. For decades, the ADHD field has called for more insight into female-specific therapy. Preliminary findings postulate that changes in sex hormones during the menstrual cycle may influence the effectiveness of psychostimulant medication. Yet, pharmacotherapeutic interventions tailored to women with ADHD remain scarce. Previously, our group showed an increase in mood symptoms in the premenstrual week in women with ADHD. Premenstrual worsening of depressive and ADHD symptoms represent a treatment challenge. In our adult ADHD clinic, we noted several women describing exacerbation of their ADHD and depressive symptoms in the premenstrual week and/or insufficient effect of their established dosage of psychostimulant. We responded to the need expressed by these women by increasing their stimulant dosage in the premenstrual week, while monitoring the response and side effects.

**Methods:**

This community case study of nine consecutive women being treated for ADHD and co-occurring conditions (including depression and premenstrual dysphoric disorder), reports our local experience of increasing the individually prescribed psychostimulant dosage during the premenstrual period. We methodically monitored the effect of this increased dosage on ADHD symptoms, mood and somatic symptoms for the following 6–24 months.

**Results:**

With premenstrual dose elevation, all nine women experienced improved ADHD and mood symptoms with minimal adverse events. Premenstrual inattention, irritability and energy levels improved, and now resembled the other non-premenstrual weeks more closely. All women decided to continue with the elevated premenstrual pharmacotherapy.

**Discussion:**

Our preliminary results demonstrate potential benefits of increasing premenstrual psychostimulant dosage in women with ADHD, experiencing premenstrual worsening of ADHD and mood symptoms. The results concur with previous findings of diminished response to amphetamines in the late luteal phase. Increased dosage may help combat premenstrual worsening of cognitive and emotional symptoms in women with ADHD, with significant clinical implications. Better management of premenstrual ADHD and mood symptoms in vulnerable women can improve treatment outcome and meet an unmet need. However, implementation should be individually explored. Further investigation of luteal phase psychostimulant dose adjustment is required for safe, optimal and individualised treatment for women with ADHD.

## 1 Introduction

Attention-Deficit/Hyperactivity Disorder (ADHD) is a common neurodevelopmental condition, characterised by lifetime difficulties in concentration, hyperactivity, and impulsivity (1). The cross-national prevalence of adult ADHD is 3.4% (2). ADHD often co-occurs with various conditions (3). The sex distribution in adulthood is close to 1:1, but girls and women with ADHD remain underdiagnosed and undertreated (4, 5).

For almost forty years, the literature has called for more insight into ADHD in girls and women (6–10) Even though scientific recognition of the impact of sex on the development of (mental) health conditions is increasing rapidly, therapeutic interventions tailored to women with ADHD remain scarce (11, 12). An expert consensus statement did not identify any differences in pharmacotherapeutic recommendations for ADHD between sexes, but did note that the menstrual phase might affect treatment response (7). Changing hormone levels might decrease effectiveness of stimulant medication (13). Further, the interaction between the menstrual cycle and ADHD might be an important missing link in clarifying ADHD in girls and women (14).

A recent systematic review could only include 4 articles and was unable to identify a clear relationship between hormonal changes and ADHD in the menstrual cycle, during pregnancy, and in the (peri-)menopausal period (15). Another systematic review identified several differences in prescription rates, efficacy and usage of ADHD medication between women and men, but also a lack of information on sex-specific pharmacokinetics and adverse effects of ADHD medication (16). The authors recommended differing dosage patterns to adjust for the menstrual phase, but remain unable to offer any additional specifics due to lack of existing evidence (16). This emphasises the need to investigate the influence of fluctuating reproductive hormones on the psychopharmacology of ADHD.

We, MJ, DW, and JK, are medical practitioners in an outpatient clinic that exclusively treats patients with adult ADHD (and co-occurring conditions). We noticed that many women with ADHD described a stark decrease in wellbeing in the premenstrual week (Box 1), with increased irritability, fatigue and a severe worsening of their mood. Some met the diagnostic criteria of co-occurring premenstrual dysphoric disorder (PMDD), of which the core symptoms include anxiety, irritability and depressed mood (17). Additionally, numerous women reported a worsening of their ADHD symptoms in the premenstrual week. A third, related complaint came from women who had been on stable doses of psychostimulant medication. They reported that their ADHD medication was either less effective or ineffective in the premenstrual week and shortly after commencement of menstruation. Some had independently increased their dosage, initially without adequate follow-up.

Box 1 Brief definitions of key terms used

**Table:** 

*Premenstrual week*	This is the week before menstruation commences. This week is also known as week 4 of the menstrual cycle. Here, we loosely use the term to signify the period in the menstrual cycle with subjective premenstrual worsening of symptoms.
*Follicular phase*	Approximately the first two weeks of the menstrual cycle, commencing on day 1 of menstruation and lasting until ovulation. Central in this phase is maturation of the ovarian follicles that contain oocytes (18).
*Luteal phase*	Approximately the last two weeks of the menstrual cycle, commencing the day after ovulation and lasting until day 1 of menstruation of the following cycle. Central in this phase is the transformation of the follicle into the corpus luteum and the preparation of the endometrium for possible implantation of a fertilised oocyte (18).
*Post-ovulatory phase*	The days immediately following ovulation, when some women experience mild “premenstrual-symptoms”. In our study, the exact moment of ovulation is unknown. We rely solely on subjective reports of ‘ovulation-like’ symptoms.

Our patients’ reports match the existing evidence. During periods of low oestrogen, increased ADHD symptoms have been described (13, 19–21). Additionally, it has been reported that women respond less strongly to psychostimulant drugs in the luteal phase (16, 22–24). Therefore, we decided to increase the dose of the prescribed psychostimulant in the premenstrual week and evaluate the effect, hoping to improve treatment and establish a foundation for further research.

## 2 Methods

We report the effect of an increased dose of psychostimulant premenstrually, as part of the treatment at the outpatient specialist clinic for ADHD in adults at PsyQ, The Hague, Netherlands. This project was carried out in accordance with the Declaration of Helsinki; safety and confidentiality were foregrounded. All women gave written consent and were aware of the experimental nature of the pharmacotherapeutic adjustments.

All patients were being treated by MJ or DW, between 09–2021 and 03–2023. All had received a psychiatric assessment at our clinic, where ADHD was diagnosed or confirmed using the DIVA-5 interview (25, 26).

All women received treatment as usual, including pharmacotherapeutic and psychological interventions aimed at minimising complaints of ADHD and co-occurring conditions, in accordance with existing treatment guidelines. Despite the fact that all women had been adhering to their prescribed psychostimulant dosage for several months, they reported that their ADHD and mood symptoms worsened premenstrually. Taking this into account, a clinical decision was made in collaboration with the women to adjust the premenstrual dosage. Other possible interventions (e.g., hormonal contraceptives or antidepressants) were deemed undesirable or insufficient. Initially, the lowest readily available dose was added. If deemed necessary by patient and/or practitioner this dose was further increased monthly, until sufficient (subjective) effect was achieved, taking side effects into account. As with treatment as usual, all dosage adjustments warranted additional check-ups to evaluate the effect and possible side effects, which we describe as reported by the women. From the time the premenstrual dosage was increased, all women had regular follow-ups for six months to two years. As we described more cases, we attempted to delineate more clearly the effect women reported on their ADHD symptoms and mood complaints. We enquired more explicitly about the fluctuation of ADHD symptoms during the menstrual cycle, as well as the presence of mood symptoms in the premenstrual week. Additionally, as we started to describe the cases more systematically, we added a 5-point Likert scale to assess the effects for both ADHD and mood complaints (1: much worse, 2: moderately worse, 3: unchanged, 4: moderately improved, 5: much improved).

## 3 Results

We included 9 consecutive women aged 22–48, who were receiving active treatment for their ADHD and co-occurring conditions. Seven patients were referred directly by their General Practitioners for diagnosis or treatment of ADHD symptoms, one by another mental health department and one by a psychiatrist in a neighbouring country. The women had a mean of 3.4 psychiatric co-occurring conditions (varying from 1–6), six had a diagnosis of PMDD and three were being treated with a selective serotonin reuptake inhibitor (SSRI) before the psychostimulant dose adjustment (Table 1).

**Table 1:** Demographic information, diagnoses and medication (history).

	Age*	Diagnoses (DSM 5)	Medication	(O)C^#^		New dosage
				Current	Past	
1	24	ADD GAD Social phobia PMDD DSPD	LDX 30 mg Melatonin 1 mg AN	IUD-	OC	LDX 40 mg
2	24	ADHD Depr-recur-remis PMDD DSPD	LDX 50 mg Melatonin 1 mg AN	No	OC	LDX 70 mg
3	26	ADHD Depr-recur-mild PMDD DSPD – RLS	LDX 70 mg Pregabalin 75 mg AN Ferr. sulphate 105mgfe 2/day	No	No	LDX 90 mg
4	48	ADHD Depr-sing-remis Panic dis. AUD-mild RLS	LDX 70 mg Seroxat 40 mg	No	OC & IUD+	LDX 100 mg
5	33	ADHD – ASD Depr-recur-remis PMDD *Ehl-Danlos – POTS*	LDX 20 mg Escitalopram 5 mg	No	OC	LDX 30 mg
6	22	ADHD – ASD Depr-recur-unspec GAD Insom – DSPD – RLS	MPH ER 72 mg Ferr. fumarate 200 mg	No	No	MPH ER 108 mg
7	30	ADHD Depr-sing-remis PMDD	DX IR 10 mg 2/day Ferr. sulphate 105mgfe 1/day	OC	OC	DX IR 15 mg 2/day
8	44	ADHD Depr-recur-moderate	DexMPH ret. 20 mg Sertraline 100 mg Atenolol 25 mg	No	No	DexMPH ret. 30 mg
9	48	ADD Sleep disorder NOS PMDD	LDX 70 mg	OC	OC	LDX 90 mg

Diagnoses: ADHD: attention-deficit/hyperactivity disorder – the combined subtype; ADD: attention-deficit/hyperactivity disorder – the inattentive subtype; AUD: alcohol use disorder; ASD: autism spectrum disorder; Depr-recur-remis/unspec: depressive disorder – recurring – in remission/unspecified; Depr-sing-remis: depressive disorder – single episode – in remission; DSPD: delayed sleep phase disorder; Ehl-Danlos: Ehlers-Danlos syndrome; GAD: generalised anxiety disorder; insom: insomnia disorder; PMDD: premenstrual dysphoric disorder; POTS: postural orthostatic tachycardia syndrome; RLS: Restless Legs Syndrome. Medication: LDX: lisdexamphetamine; MPH ER: methylphenidate extended release; DX IR: dexamphetamine immediate release; DexMPH ret: dexmethylphenidate retard; Ferr. sulphate/fumarate: ferrous sulphate/fumarate; (O) C: (oral) contraceptive; IUD+/−: intrauterine device with (+), or without (−) hormones. * At start of increased premenstrual dosage # Use of (oral) contraceptives at time of increased dosage (current) and in the past.

The exact moment in the cycle when the increased dosage was required (commencement: 3–10 days before; until: 0–5 days after start of menstruation), the duration of using the higher dose (between 3–10 days total) and the amount with which the original dose was increased (range 30–50%), varied between cases. Follow-up ranged from 6–24 months. We summarise the reasons for increasing the premenstrual dosage and the effects thereof per patient (Tables 2, 3).

**Table 2:** Summary of response to dose increase.

	Age*	Regular dose	Premenstrual dose	Increase	Effect ADHD^a^		Effect mood^a^		Since^b^	Duration^c^	Y/N^d^
					1st	Following	1st	Following			
1	24	LDX 30 mg	LDX 40 mg	30%	–	**4**	–	5	11–2022	10	Y
	*Summary:*	Improved ADHD symptoms, mood stabilisation; and reduced irritability									
2	24	LDX 50 mg	LDX 70 mg	40%	4	**4,5**	5	4,5	02–2023	7	Y
	*Summary:*	Improved ADHD symptoms, emotional regulation; and reduced intensity of emotional experiences									
3	26	LDX 70 mg	LDX 90 mg	30%	–	**4**	–	4	01–2023	8	Y
	*Summary:*	Improved ADHD symptoms, better focus, productivity; and mood stabilisation									
4	48	LDX 70 mg	LDX 100 mg	45%	4	**5**	–	4,5	03–2023	6	Y
	*Summary:*	Initial difficulty implementing the higher dose, but subsequently improved concentration, emotional control, and mood									
5	33	LDX 20 mg	LDX 30 mg	50%	4	**4**	4	4,5	02–2023	7	Y
	*Summary:*	Better focus, increased productivity, reduced irritability; and fewer mood swings									
6	22	MPH ER 72 mg	MPH ER 108 mg	50%	–	**5**	–	4	11–2021	22	Y
	*Summary:*	Improved ADHD symptoms, reduced hypersensitivity, better focus, and mental clarity									
7	30	DX IR 10 mg 2/day	DX IR 15 mg 2/day	50%	–	**4**	–	4	09–2021	24	Y
	*Summary:*	Better self-management, improved mood; and reduced binge eating									
8	44	DexMPH ret. 20 mg	DexMPH ret. 30 mg	50%	4	**4**	5	4	03–2022	18	Y
	*Summary:*	Improved focus, productivity and mood; with no change in physical symptoms									
9	48	LDX 70 mg	LDX 90 mg	30%	4	**4**	4	4	03–2023	6	Y
	*Summary:*	Better focus, concentration; improved mood and energy levels									

Medication: LDX: lisdexamphetamine; MPH ER: methylphenidate extended release; DX IR: dexamphetamine immediate release; DexMPH ret: dexmethylphenidate retard. * At start of increased premenstrual dosage.

a Scored on a 5-point Likert-scale (1: very much worsened; 2: moderately worsened; 3: no change; 4: moderately improved; 5: much improved) after the first month of using the higher dose (1st), and after multiple months of using the higher dosage (Following).

b Date from which the women have been using the increased premenstrual dosage.

c How many months have women used the higher dose at time of final text revisions (09–2023).

d Do women wish to continue using the higher premenstrual dose: yes/no (Y/N).

**Table 3:** Summarised self-reported symptoms and symptom reduction.

Pt	Premenstrual symptoms	Symptom reduction
1	Increased ADHD symptoms; clumsiness Irritability Crying Low and labile mood Decreased energy Heightened anxiety	Improved ADHD symptoms Mood stabilisation Reduced irritability Less exhaustion
2	Dramatic increase in ADHD; chaos and more mistakes Dramatic increase in depressive symptoms Irritability Hypersensitivity Decreased energy	Marked improvement in ADHD symptoms; fewer errors and less chaos. Improved emotional regulation Reduced intensity of emotional experiences Improved energy level
3	Less effect of ADHD medication Decreased focus and productivity Lower mood with mood swings Stark decrease in energy Feeling worthless, more sensitive, irritable and emotional	Improved ADHD symptoms; better focus, productivity Mood stabilisation Increased productivity Improved energy levels More resilience
4	Increased ADHD symptoms; making errors, forgetful, clumsy, chaotic and ‘brainfog’ Many mood swings Irritability Anxiety, insecurity and depressed mood	Improved ADHD symptoms; better concentration, less clumsy, more alert More emotional control, but internally still labile Less agitated Improved mood Clear improvement in energy level
5	Decrease in focus and concentration Poor energy Loss of interest and grumpiness Unable to keep up with tasks.	Better focus, increased productivity More calm, with decreased anxiety Reduced irritability Fewer mood swings Marked improvement in energy level
6	Increased ADHD symptoms; ‘brainfog’, forgetfulness and mental chaos Lower mood Sensory and environmental hypersensitivity Less energy	Excellent improvement in ADHD symptoms; better concentration, mental clarity, less ‘brainfog’ Reduced hypersensitivity More stable mood Less anxiety
7	Increased ADHD symptoms; disorganisation, poor focus Lower mood Irritable and impatient Lower energy	Better self-management; better focus and concentration Improved depression and anxiety Fewer emotional meltdowns Less irritability More energy Reduced binge eating
8	Increased ADHD symptoms; poorer planning and making decisions Lower mood Anxiety Irritability	Improved focus, productivity, planning and organisation Less avoidant Better mood and emotionally much less volatile Tiredness remained
9	Increased ADHD symptoms; forgetfulness and ‘brainfog’, poor planning and sense of time Depressed mood and emotionally volatile Irritable and impatient	Much better focus and concentration, less ‘brainfog’ and feeling more present Improved mood; less depressed, angry and volatile Less agitated Better energy levels

### 3.1 Patient 1 (24 yrs)

#### 3.1.1 Reasons

In the premenstrual week, she described increased ADHD symptoms: less focus, more chaos and more trouble keeping up with necessary duties. She also suffered from increased irritability, low mood, more mood swings, more anxiety and decreased energy. She described herself as more “snappy,” being quite reactive and crying often. Generally, she considered herself a clumsy person, which worsened premenstrually.

Physically, she described premenstrual fatigue, general malaise, mild abdominal cramps and severe premenstrual backache. Her sleep worsened with an occasional night of total insomnia.

Her dose was increased from lisdexamphetamine 30 mg to 40 mg daily (*circa* 30% increase), 3–4 days before and the first 2–3 days of her menstruation.

#### 3.1.2 Effects

Her report was positive from the first month of increased dosage. She described her experience with the higher dose as “much more smooth,” recognising less increase of her ADHD symptoms, exhaustion and irritability around her menstruation. While she would still feel defensive, she was more in control of how she reacted to emotions and could choose not to react. This resulted in fewer arguments. She was able to do necessary tasks, like cooking, and was better able to keep her routine. Her mood and irritability remained more consistent throughout the month. She could still feel down, irritable and “snappy” upon waking up, but this would fade when the medication started working. She also reported having better motor control. Physically, she felt less tired and had less bodily pain. Her sleep did not improve with the higher dose, but she was able to get through the following day with more energy.

She did not report increased side effects.

### 3.2 Patient 2 (24 yrs)

#### 3.2.1 Reasons

After quitting the oral contraceptive, she noticed a dramatic increase in ADHD and depressive symptoms in the week before and the first couple of days of her menstruation. In particular, she described an increase in chaos, irritability and making more mistakes in the premenstrual week. She reported experiencing everything very intensely, being hypersensitive to small triggers and easily angered. Additionally, she was feeling more down and had decreased energy.

She did not report any physical complaints.

Her dose was increased from lisdexamphetamine 50 mg to 70 mg daily (*circa* 40% increase), in the second phase of her menstrual cycle, about 10 days before menstruation.

#### 3.2.2 Effects

She noticed a marked improvement in her ADHD symptoms from the first month. She made fewer errors and experienced less chaos. She had better focus and concentration. The intensity of her emotional experiences and reactions was reduced, she could handle everything much better and her irritability was less. Her mood and energy level both improved. The difference between her premenstrual week and the other weeks was less marked.

#### 3.2.3 Notes

While fasting during Ramadan, she was able to continue with the increased dosage, without experiencing increased side effects.

She did not report increased side effects.

### 3.3 Patient 3 (26 yrs)

#### 3.3.1 Reasons

In the week before, and first days of her menstruation, she noticed less effect of her ADHD medication, a stark decrease in energy and an increase in depressive symptoms. In particular, she experienced decreased focus and productivity. She struggled with tasks that were boring or necessitated much work, which made it very difficult to work from home and led to task-oriented anxiety. She also reported more prevalent mood swings, feeling worthless, more sensitive, irritable and emotional.

Physically, she had fatigue and severe stinging abdominal pain in the premenstrual period, which was followed by abdominal cramps as her menstruation commenced.

Her dose was increased from lisdexamphetamine 70 mg to 90 mg daily (*circa* 30% increase), for 4–5 days before menstruation.

#### 3.3.2 Effects

From the first month of increased dosage, she noticed a strong improvement in the ADHD symptoms. Working from home was easier, she could focus more and for longer periods. She was motivated to start and complete tasks that she would otherwise avoid. She worked without “stressing herself out” and her productivity increased. She could regulate her emotions better. Her mood swings and irritability remained, but she could move on from them more quickly, recognising their cause. She was still “all over the place,” but could distance herself from this more easily, relate it to hormonal fluctuations and let it go more quickly. With the higher dose, she completed her day with “a little bit of struggle, rather than a lot” and got through the week with more resilience. She could “take the punches better.” She had more patience with herself and others. She noticed an improvement in her mood and energy level. She reported feeling more alert, less anxious and stressed. Usually, she did not take her medication during the weekends, but in the premenstrual phase she did.

#### 3.3.3 Notes

In May 2023, after 3 months of using the increased premenstrual dosage, it was decided to commence treatment with an SSRI because her depressive symptoms remained debilitatingly present, approximately 3 weeks every month. Initially, she had strongly opposed starting an SSRI, so we agreed to try increasing her psychostimulant dosage first. With the additional SSRI her mood improved and stabilised, and her stress decreased further. She persisted with the higher premenstrual dose of lisdexamphetamine in combination with escitalopram 20 mg.

She noted a slightly stronger rebound effect and became aware of her caffeine intake, but did not report other additional side effects.

### 3.4 Patient 4 (48 yrs)

#### 3.4.1 Reasons

In the premenstrual week she described increased ADHD symptoms: making more errors, being much more forgetful, and more clumsy and chaotic. She reported being emotionally labile and experiencing many mood swings. She would react to her surroundings more, engaging in conflict or feeling angry at everything. She described being confused, all over the place and experiencing “brainfog.” Her irritability increased and her energy level was much lower, her mood more down. She would feel much more anxious, linger in negative feelings for longer periods of time and felt more insecure.

Physically, she described fatigue, tender breasts and a distinct worsening of Restless Legs Syndrome in the premenstrual week, which negatively influenced her sleep quality.

Her dose was increased from lisdexamphetamine 70 mg to 100 mg daily (*circa* 43% increase), 10 days before menstruation.

#### 3.4.2 Effects

She noticed an improvement in her ADHD symptoms, from the third month of the increased dosage. The first two months, she struggled to remember to increase her dose and to determine when the optimal moment was. After implementing the higher dose successfully, she described being less clumsy, bumping into things less. Her concentration was better and she felt more alert, with less “cotton wool in her head.” She understood and kept up with her schema therapy better. She reacted less emotionally, with a more delayed, or less intense response. The higher dose “took the edge off,” she would feel angry, but would not start shouting immediately. She noticed a clear improvement in her energy level, which impacted her mood in a positive way. Even though she still described being agitated, this was less than it had been with her regular dosage. However, she reported that the level of emotionality had not changed.

#### 3.4.3 Notes

Because of persisting complaints, which may be related to (peri-)menopause, she is considering additional hormone replacement therapy.

She did not report increased side effects.

### 3.5 Patient 5 (33 yrs)

#### 3.5.1 Reasons

In the premenstrual week she noticed a decrease in focus and concentration and was unable to keep up with household chores, let alone work. In addition, she described a stark decrease in energy. She felt more down and experienced a loss of interest. She reported being more irritable, easily angered and would become grumpy more quickly.

Physically, she described premenstrual fatigue, migraines, headache and tender breasts.

Her dose was increased from lisdexamphetamine 20 mg to 30 mg daily (50% increase), for 3 days before her menstruation.

#### 3.5.2 Effects

She described a big difference from the first month of the increased dosage. She reported more focus and was able to concentrate for longer. Her productivity increased and it was easier to get started on tasks, even the unpleasant ones; she “just did them.” This brought a sense of calm and decreased anxiety, because she managed to stay on top of things more and keep up with household chores. She reported a marked improvement in her energy level, which influenced her mood in a positive way. In general she reported less irritability and fewer mood swings. As she was less tired, she was able to handle her physical complaints better. In addition, she noticed an effect of the higher dose on her physical complaints and experienced less headache and migraines. Conversely, she noted more eczema, dry skin and allergies, which could also be related to the season (Spring). She reported more muscle weakness and fluid retention, possibly related to her known sensitivity to hormone fluctuations and weather changes.

#### 3.5.3 Notes

Her slow metaboliser status (decreased CYP2C19 and CYP2D6 function) was already known, therefore she required lower dosing.

The higher premenstrual dose made it easier for her to go into ‘overdrive’ and exhaust herself, which was a known pitfall for her and which required extra attention after increasing the dose.

She reported that she had to avoid caffeine completely and be careful not to exhaust herself, but did not note any additional side effects.

### 3.6 Patient 6 (22 yrs)

#### 3.6.1 Reasons

In the premenstrual week she described increased ADHD symptoms: being more forgetful and chaotic, with “brainfog” and reduced mental clarity. She described her mood as sad, hopeless, more irritable as well as hypersensitive to triggers in her environment. Specifically, she was very frustrated with small, generally “insignificant” things which she could usually tolerate. She pulled back socially and described increased “sensory distress.” Premenstrual sensory hypersensitivity resulted in difficulty with smells, textures, lights and noises. She therefore avoided very loud or bright spaces, limited her social activities, and attendance at university classes, and prepared rapid and very simple meals with few ingredients. She also had increased hypersensitivity to certain fabrics. She summarised her premenstrual state as diverting all the energy she usually used for her daily life to managing her own body and self.

Physically, she described less energy, appetite changes such as extremely hungry or no appetite, a sensation of bloating. Her bodily symptoms included aches and pains, fatigue, poor sleep schedule, resulting in delay of her sleep onset. Her sleep onset time was between 2 or 3 a.m. instead of her habitual time between 10:30 and 11:30 p.m.

Her dose was increased from methylphenidate 72 mg daily to 108 mg daily (50% increase), for 3 days premenstrually and for the 5 days of menstruation.

#### 3.6.2 Effects

She noticed “excellent” improvement in ADHD symptoms from the first month. Premenstrually and during her menstruation, she noticed her mood being more stable with less anxiety, and fewer appetite changes. She had better sleep with earlier sleep onset. She was more productive with improved focus. Her bodily hypersensitivity was reduced. She described how the increased dosage allowed her to focus less on bodily discomfort with more mental space for concentrating on necessary tasks. She had more mental clarity and less brainfog.

She did not report increased side effects.

### 3.7 Patient 7 (30 yrs)

#### 3.7.1 Reasons

In the premenstrual week she noted increased ADHD symptoms: more disorganisation, inability to complete tasks and poor focus in conversations. Her mood was lower, more irritable and impatient.

Physically she tended to have irregular eating habits and would binge in response to stress premenstrually. Her energy level was also lower.

Her dose was increased from dexamphetamine immediate release 10 mg once to twice daily to 15 mg once to twice daily (50% increase), for 7 days premenstrually.

#### 3.7.2 Effects

She described a marked improvement in ADHD symptoms from the first month. She felt more in control at work as she could structure her day better, was more able to delegate, plan and had better self-management. She had more energy for household chores, with minimal procrastination. With better focus and concentration, she no longer felt the drive to be continuously busy, felt more rested and peaceful. Her depressive and anxiety symptoms improved, with less irritability. She took criticism less personally. With normal life stresses, she no longer felt overwhelmed, and “emotional meltdowns” ceased. Her eating patterns became more regular and premenstrual bingeing stopped. She could fall asleep earlier.

She did not report increased side effects.

### 3.8 Patient 8 (45 yrs)

#### 3.8.1 Reasons

In the premenstrual week she noted increased ADHD symptoms: difficulty planning ahead and making decisions, feeling less in control, poor focus. Premenstrually, she described her mood as lower, anxious and more irritable.

Physically she described the following mild symptoms: nausea, breast sensitivity, headache, irregular bowel habits (diarrhoea and constipation), abdominal cramps, bloated feeling.

Her dose was increased from dexmethylphenidate retard 20 mg daily to 30 mg daily (50% increase), for 7 days premenstrually.

#### 3.8.2 Effects

She described a moderate difference in her ADHD symptoms from the first month. Her focus, productivity, ability to start with and switch tasks, planning, organisation and attention improved but were not completely optimal. She no longer needed deadlines to complete tasks and was less avoidant. Her mood and irritability improved significantly and she was emotionally much less volatile. She was less anxious and felt more in control. There was no change in her physical symptoms and she remained tired.

She did not report increased side effects.

### 3.9 Patient 9 (48 yrs)

#### 3.9.1 Reasons

In the premenstrual week she noted increased ADHD symptoms: she described her mind as less clear, forgetful, “brainfog,” had little perspective and poorer concentration. She had difficulty planning ahead, poorer sense of time, felt less in control, poor focus and battled to make decisions. Emotionally she was very depressed, emotionally volatile and angry with mood swings, irritability and impatience.

Physically she described premenstrual nausea, poor energy levels and constant tiredness. Everything felt like an effort.

Her dose was increased from lisdexamphetamine 70 mg to 90 mg daily (*circa* 29% increase), for 7 days premenstrually.

#### 3.9.2 Effects

She described an improvement in ADHD symptoms from the first month with much better focus, less “brainfog,” improved concentration, less distractibility and felt more present in situations. She was more energetic, productive and less avoidant of tasks. Her mood was less depressed, angry, volatile and irritable. Mood swings and agitation improved. She could begin road running again.

She did not report increased side effects.

## 4 Discussion

In this case study, we investigated the impact of increasing premenstrual psychostimulant dosage on nine consecutive adult women with ADHD. These participants reported premenstrual worsening of ADHD and mood symptoms. Our decision to increase psychostimulant dosage during the premenstrual week stemmed from other patients in our ADHD clinic, who independently increased their dosage without proper monitoring. Our approach involved raising the psychostimulant dosage during the premenstrual week and tracking its effects on ADHD, mood, and somatic symptoms over subsequent months, while continuing treatment as usual. To the best of our knowledge, this constitutes the only study of its kind published so far. As is common in adult ADHD (7, 27), these women were diagnosed with ADHD and several co-occurring conditions (Table 1). All had been taking stable psychostimulant treatment for several months, but expressed dissatisfaction with its efficacy during the late luteal phase, with consequential worsening of ADHD and mood symptoms. Methodically elevating the prescribed psychostimulant dosage during the premenstrual phase yielded positive results, with participants noting improvements in ADHD and mood symptoms. Additionally, they could better deal with their somatic symptoms, which improved for some.

Before the premenstrual dosage increase, the women in our study experienced the premenstrual phase as severely invalidating. They reported decreased focus, concentration, productivity, and “brain fog.” They also described compromised self-control, leading to heated arguments, binge-eating, and impulsive behaviours. The aftermath of this phase included feelings of regret, shame, and a perceived lack of control over their actions. This debilitating pattern repeated itself every month and hindered women in finding and maintaining a healthy, balanced life. Unfortunately, the experiences of the patients described here appear to be quite common (13, 28, 29). They align with a growing framework exploring the interplay between hormonal fluctuations and ADHD symptoms (15). This underscores the importance of such investigations for effective treatment for women with ADHD.

Despite variations in symptomatology, age, co-occurring conditions, and type of psychostimulant, all nine women experienced and scored a positive change in premenstrual mood and ADHD symptoms with the increased dosage and wished to continue using it (Table 1). All reported improvement within the first month, except for one participant (Pt 4) who initially struggled with adherence to and timing of the increased dosage. Improvements in ADHD symptoms that were consistently noted were: better concentration, focus, productivity, and a greater ability to regulate or manage emotions. Additional side effects were minimal to absent for all women. Premenstrual mood improved for all patients, with eight reporting reduced irritability (Pts 1,2,3,5,6,7,8,9), seven describing improved energy levels (Pts 1,2,3,4,5,6,9), six experiencing decreased agitation (Pts 1,2,4,6,8,9), and four reporting less anxiety (Pts 3,5,6,7). After dose increase, six women noted fewer mood swings or less impact thereof (Pts 1,3,5,6,8,9) and four women explicitly reported feeling more in control of their emotional reactions (Pts 1,3,6,8). An additional six described being less emotionally volatile or “reactive” (Pts 2,4,6,7,8,9). In general, many described a “normalisation” of premenstrual symptoms, and less pronounced distinctions between premenstrual and non-premenstrual weeks. Notably, some women observed improvement of premenstrual physical symptoms (Pts 1,5,6,7,9) or ability to tolerate these (Pts 3,5,6).

In healthy women, sex hormones are known to influence neurotransmitters, like dopamine (30) and serotonin (31–33). Thus, fluctuations in reproductive hormones during the menstrual cycle are thought to impact emotional states, mood disorders (34–38) and cognition (14, 39). A recent systematic review by Dubol et al. concluded that brain structure and reactivity are affected by hormonal fluctuations, which impact negative affect and cognition (40). Sacher et al. reviewed the existing neuroimaging studies and found that changes across the menstrual cycle influence the reaction to emotional stimuli and rewards. Amongst other effects, cyclical hormone fluctuations appear to interact with dopaminergic transmission (39). In healthy women, the interaction between sex hormones and neurotransmitters is also believed to influence ADHD symptoms (14, 19, 41). Low oestrogen phases correlate with increased ADHD symptoms. Young women without ADHD display heightened ADHD symptoms, particularly high trait impulsivity, during both early follicular and early luteal, or post-ovulatory phases (19). Focussing on menopausal women, Shanmugan et al. linked oestrogen to working memory, sustained attention and executive functions (41) and showed that lisdexamphetamine improved executive functioning in healthy menopausal women with executive difficulties (20). Anticipated work of Wasserstein et al. seems to solidify the relation between ADHD (symptoms) and menopause (21).

Other research findings explicitly link the effect and (ab)use of psychostimulants (particularly amphetamines) to changing levels of progesterone and oestrogens throughout the menstrual cycle (23, 24, 42, 43). In the luteal phase, women appear to respond less strongly to psychostimulant drugs (16, 22–24). In young women, fluctuating oestrogen levels may also influence the effectiveness of stimulant medications. A small study in 16 healthy women by Justice et al. showed that the effects of dextro-amphetamine (15 mg orally) were greater during the follicular phase than the luteal phase (23). During the follicular phase, subjects reported feeling more “high,” “energetic and intellectually efficient” after taking dextro-amphetamine, than during the luteal phase. While oestrogen seems to aid in the effectiveness of stimulants, progesterone likely decreases it (23). These results were replicated by another small study showing that oestrogen and progesterone levels may impact on the subjective euphoric and stimulating effects of dextro-amphetamine in healthy women who are not affected by ADHD (24).

The findings in healthy women regarding hormone interaction with neurotransmitters raise questions about their relevance in women with ADHD. Low oestrogen phases might exacerbate cognitive and mood symptoms in these women. This corresponds with a case study showing worsened ADHD and mood symptoms premenstrually in a young woman with ADHD (13). Our participants’ experiences align with existing evidence of increased ADHD (12–14, 19, 41), coupled with decreased response to psychostimulants during low oestrogen phases (16, 22–24). Notably, six out of nine women exhibited co-occurring PMDD, mirroring our group’s previous findings of increased prevalence and severity of PMDD symptoms in a cohort of women with ADHD (28). Interestingly, the women in this cohort also described an increased prevalence of postpartum depression and peri-menopausal symptoms. These are additional periods in women’s reproductive lives characterised by low oestrogen levels (28). The hypothesis emerges that in the luteal phase, when oestrogen levels fall, dopamine neurotransmission is further compromised in women with ADHD, leading to an exacerbation of their low mood and ADHD symptoms. This may explain the perceived ineffectiveness of the previously established psychostimulant dosage. Therefore, an increased psychostimulant dose may help alleviate worsening ADHD and mood symptoms in the premenstrual phase.

### 4.1 Clinical implications

We present a promising, relatively quick and easy intervention for the prevalent and debilitating issue of premenstrual worsening of ADHD and mood symptoms for women with ADHD. All women reported improvement with minimal increase in side effects. Healthcare professionals should initiate the conversation about this topic. Women may lack awareness of the far-reaching implications of their cyclical pattern, or feel embarrassed discussing it. Women with ADHD are particularly vulnerable: without treatment, they may lack the necessary overview and sense of timing needed to describe the impact of hormonal fluctuations on their mood and well-being (28). Failing to consider the menstrual cycle can result in sub-optimal treatment. Women may adjust dosage themselves.

The clinical implications of our findings are summarised in Box 2. Personalised dosing and timing adjustments of the psychostimulant are crucial, necessitating careful monitoring and cycle awareness. All women emphasised the increase in their energy level. However, clinicians should be cautious of women exhausting themselves and monitor, counsel and adjust treatment as necessary. Increased psychostimulant dosage should not replace SSRI treatment for depressive symptoms, nor oral contraceptives for physical complaints. If mood is insufficiently improved with increased psychostimulant dosage, addition of an SSRI can be beneficial and complement dose increase.

Box 2 Clinical implications of increased premenstrual psychostimulant dosage

- May help control premenstrual worsening of ADHD and mood
- Consistent improvements in focus, energy, productivity and mood
- Cycle awareness is essential: *PMDD calendars or applications may help*
- Dosing and timing of increase should be individually determined
- Monitoring and adjustments should be personalised
- Appears to be valid for several types of psychostimulants
- Additional side effects are minimal or absent
- Satisfactory effectiveness: all women were motivated to continue

Please take note: Increased premenstrual dosage does not replace SSRI for depressive symptoms, or OC for somatic complaints, but may function complementarily.

## 5 Limitations and strengths

While this study is the first (to our knowledge) to describe the beneficial effect of increased premenstrual psychostimulant dosage for women with ADHD, it does have limitations. Firstly, we present a very small number of patients in a descriptive manner and lack a control group. However, these limitations are inherent to case study design. Secondly, we adjusted our assessment of the effects of our intervention, as we proceeded with describing more cases. However, as we optimised our assessments by adding a Likert-scale, our initial results were confirmed, strengthening their internal validity. Thirdly, the women had several co-occurring conditions, which may have influenced their response to the psychostimulant dose increase, but simultaneously reflects daily practice. Finally, we acknowledge that the intervention offered here requires careful tracking of the menstrual cycle, which may be difficult for women with ADHD, and challenging for those with irregular cycles.

To strengthen our approach, we included consecutive patients, offered detailed descriptions of our nine cases and attempted to provide sufficient demographic, diagnostic and therapeutic background information, conform the JBI critical appraisal tool for case series (44).

### 5.1 Future research

It is important to replicate the findings of this case study in larger trials; ideally randomised, double-blind clinical trials, including a placebo arm, with a long(er) follow-up. A more detailed assessment of the interaction between ADHD (symptoms) and fluctuating (female) sex hormones is warranted. It would be interesting to extend the study of psychostimulant medication in postnatal and (peri-)menopausal women. Finally, we suggest examining the role of non-stimulant medications, perhaps even similar dosage adjustments, in the treatment of the ADHD/PMDD (symptom) combination. In general however, we argue that all future research concerning ADHD in women should at least take the menstrual cycle, or hormonal fluctuations in general, into consideration.

## 6 Conclusion

This case study demonstrates the potential benefits of increasing premenstrual psychostimulant dosage for managing premenstrual worsening of symptoms in women with ADHD. Improvements in ADHD symptoms, mood stabilisation, emotional control, and productivity were reported, with no worsening of side effects. These findings align with the concept of hormonal fluctuations impacting neurotransmitter function, affecting emotions and cognition. Furthermore, hormonal changes during the menstrual cycle may influence psychostimulant medication effectiveness. While preliminary, these results suggest that elevating premenstrual psychostimulant dosage might offer an efficient option for alleviating ADHD and premenstrual mood symptoms in women with ADHD, contributing to their overall wellbeing. Further research is needed in this important field to validate these findings and establish guidelines for personalised treatment plans based on menstrual cycle phases.

## Data availability statement

The datasets presented in this article are not readily available because we are describing clinical findings and patient privacy will be protected, further inquiries can be directed to MJ, m.dejong@parnassia.nl.

## Ethics statement

Ethical approval was not required for the studies involving humans because the intervention described here was offered in the context of regular clinical treatment, after careful discussion with all patients, in accordance with the ethical principles of the Parnassia Group. All patients gave written informed consent for their clinical data to be reported and published. The studies were conducted in accordance with the local legislation and institutional requirements. Written informed consent for participation was not required from the participants or the participants’ legal guardians/next of kin in accordance with the national legislation and institutional requirements because all patients had a therapeutic relationship with either MJ or DW. During the course of the therapeutic intervention and before reporting the data, written informed consent was obtained from all patients to publish clinical findings.

## Author contributions

MJ: Conceptualization, Investigation, Writing – original draft. DW: Conceptualization, Investigation, Writing – original draft. EA: Writing – review & editing. AB: Writing – review & editing. JK: Supervision, Writing – review & editing.
